# Research Progress on Epigenetics of Diabetic Cardiomyopathy in Type 2 Diabetes

**DOI:** 10.3389/fcell.2021.777258

**Published:** 2021-12-24

**Authors:** Jianxin Deng, Yunxiu Liao, Jianpin Liu, Wenjuan Liu, Dewen Yan

**Affiliations:** ^1^ Department of Endocrinology, Shenzhen Second People’s Hospital, The First Affiliated Hospital of Shenzhen University, Health Science Center of Shenzhen University; Shenzhen Clinical Research Center for Metabolic Diseases, Shenzhen, China; ^2^ Health Science Center of Shenzhen University, Shenzhen, China

**Keywords:** diabetes, diabetic cardiomyopathy, epigenetics, hyperglycemia, methylation

## Abstract

Diabetic cardiomyopathy (DCM) is characterized by diastolic relaxation abnormalities in its initial stages and by clinical heart failure (HF) without dyslipidemia, hypertension, and coronary artery disease in its last stages. DCM contributes to the high mortality and morbidity rates observed in diabetic populations. Diabetes is a polygenic, heritable, and complex condition that is exacerbated by environmental factors. Recent studies have demonstrated that epigenetics directly or indirectly contribute to pathogenesis. While epigenetic mechanisms such as DNA methylation, histone modifications, and non-coding RNAs, have been recognized as key players in the pathogenesis of DCM, some of their impacts remain not well understood. Furthering our understanding of the roles played by epigenetics in DCM will provide novel avenues for DCM therapeutics and prevention strategies.

## Introduction

According to the World Health Organization, over 422 million people (∼6% of the global population) have been diagnosed with diabetes, a number that continues to increase each year (Sneha and Gangil, 2019). Diabetes that is accompanied by various other syndromes represents the leading cause of morbidity and mortality across the global. Clinical parameters such as poor glycemic control and insulin response to diabetes contribute to heart problems (Matheus et al., 2013). Recent studies have found that cardiovascular patients with diabetes have a worse prognosis than those without diabetes. The mortality due to cardiovascular disease in diabetic patients is s3-5 times higher than that in the general population (Rawshani et al., 2017; Fedeli et al., 2019). Diabetic cardiomyopathy (DCM), a cardiovascular complication associated with diabetes, is a severe form of cardiac dysfunction caused by changes in the structure and contractility of the myocardium (Dong et al., 2017). Approximately 12% of diabetic patients eventually develop severe heart failure (HF) and often die due to DCM (Trachanas et al., 2014). Additional comorbidities in this population of patients include hypertension, obesity, dyslipidemia, and vascular disease (Lorenzo-Almoros et al., 2017).

## Diabetic Cardiomyopathy

**FIGURE 1:**
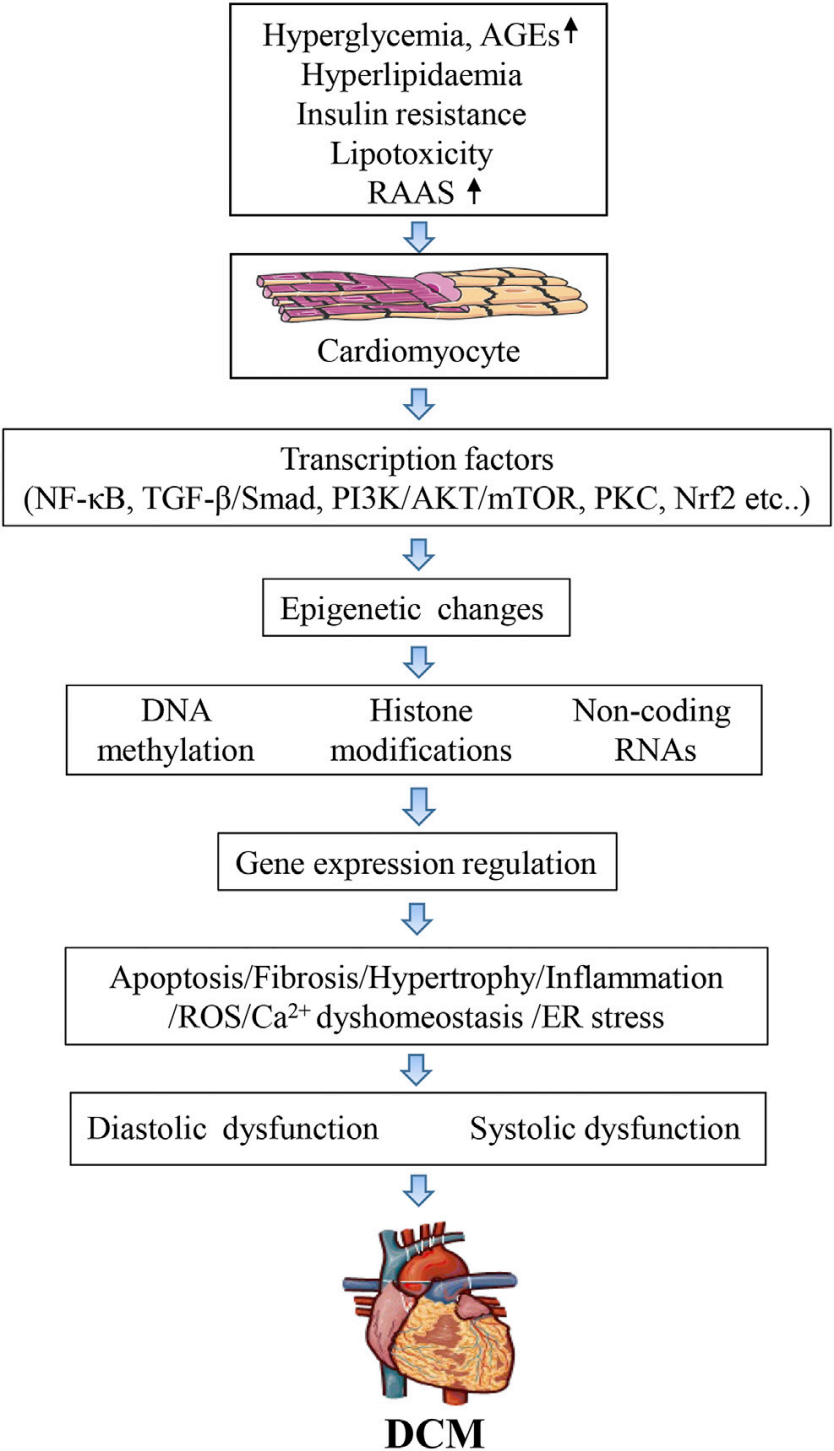
Mechanisms of epigenetic on diabetic cardiomyopathy.

DCM is a serious complication of the myocardium of diabetic patients characterized by ventricular dilation and hypertrophy, diastolic dysfunction, decreased or preserved systolic function, and reduced ejection fraction, with no accompanying coronary artery disease or hypertension (Rubler et al., 1972). The diagnostic criteria for DCM include left ventricular diastolic dysfunction, left ventricular ejection fraction (EF) reduction, pathological left ventricular hypertrophy, and interstitial fibrosis (Fontes-Carvalho et al., 2015). Despite these devastating effects, there are still no effective and specific tools to diagnose DCM. Numerous studies over the past decades have highlighted the complexity of DCM pathogenesis, identifying that multiple molecular mechanisms synergistically damage cardiomyocytes and impair heart function. The metabolic environment associated with diabetes (e.g., high blood sugar, increased circulating fatty acids and triglycerides, hyperinsulinemia, and increased inflammatory cytokines, that activate transcription factors and change various molecular pathways in cardiomyocytes), reduces myocardial contractility and causes cardiomyocyte dysfunction, cell damage, and death. Thus, the mechanisms underlying DCM pathogenesis are extremely complex and involve changes in 1), signal transduction (insulin signal, renin-angiotensin signal); 2), metabolism (glucose and lipid metabolism), calcium homeostasis, and mitochondrial function; 3), gene regulation (activation of transcription factors and epigenetic mechanisms); 4), post-translational modification of signaling proteins; 5), homeostasis of cellular processes such as apoptosis, autophagy, and endoplasmic reticulum stress (Figure 1).

Although DCM pathogenesis is multifactorial, hyperglycemia (Singh et al., 2018; Evangelista et al., 2019) is still considered a significant driver of myocardial damage (Seferovic and Paulus, 2015). Type 1 and type 2 diabetes(T2D) affect both systolic and diastolic function, and evidence from animal and the human study suggests that DCM occurs in T2D, affecting heart function and morphology and increasing the risk of HF (Holscher et al., 2016; Zhang et al., 2018; Yang et al., 2019). External stimuli or environmental factors often trigger this change. For example, changes in blood glucose levels can stress intracellular pathways that activate various transcription factors that alter global gene expression. Changes in the activity of transcription factors can ultimately lead to structural changes in the body (Lee et al., 2004). Dysregulation of coding and non-coding genes can lead to various cardiovascular diseases, including DCM (Dorn and Matkovich, 2008; Khraiwesh et al., 2010), and recent studies have implicated epigenetic and microRNAs (miRNAs) in this process. This review focuses on the role of epigenetic changes in DCM.

## Epigenetics

Epigenetics refers to heritable changes in gene expression that do not involve changes in nucleotide sequence. Epigenetic phenomena include DNA methylation, genomic imprinting, maternal effects, gene silencing, nucleolar dominance, dormant transposon activation, and RNA editing. Through these changes, the epigenome provides information about the structure of crucial functional elements that regulate gene expressions, such as methyl-labeled DNA and histones, and the interaction between the distal portions of chromatin (Romanoski et al., 2015). Epigenetic mechanisms such as DNA methylation, chromatin remodeling, and histone modifications regulate gene expression in response to change in the cellular microenvironment. These processes also play essential roles in HF (Egger et al., 2004; Mateo Leach et al., 2010), including HF that results from DCM. Although these epigenetic modifications can induce chronic disruptions in gene expression, recent studies have shown that complex interactions between genes and the environment may play an essential role in the pathogenesis of DCM and can be manipulated through diet, exercise, and drug interventions (Pepin and Wende, 2019).

## DNA Methylation

**FIGURE 2:**
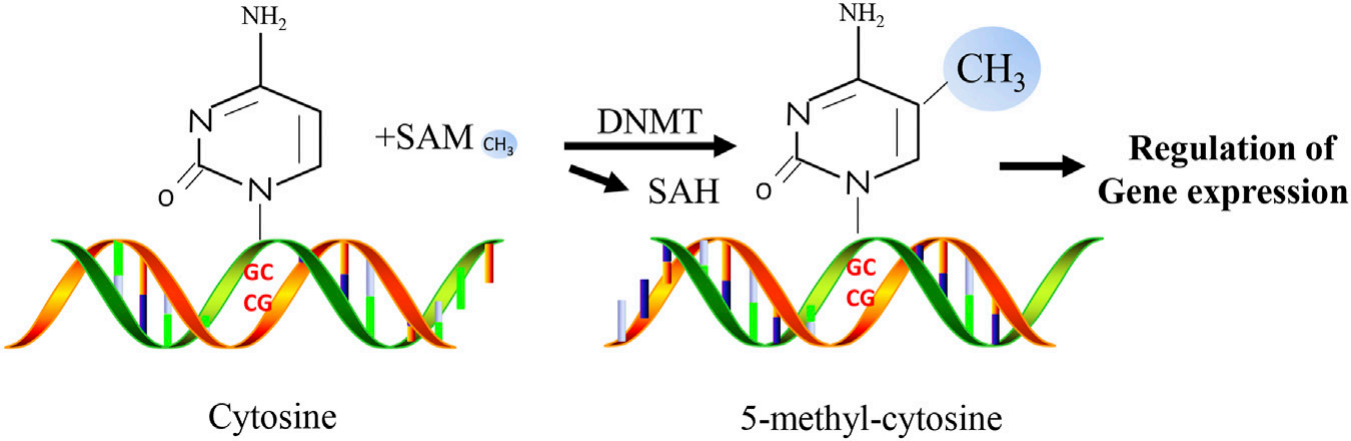
Simplified overview of molecular mechanisms of DNA methylation.

DNA methylation is an important epigenetic mechanism and is one of the most widely studied epigenetic markers (Ambrosi et al., 2017). As a significant mechanism of epigenetic regulation in mammalian cells, DNA methylation is essential for mammalian development and plays a critical role in gene silencing, genomic stability, and parental imprinting during mitosis (Smith and Meissner, 2013). DNA methylation refers to the biochemical reaction of adding methyl groups to DNA nucleotides (cytosine or adenine) which is performed by DNA methyltransferases (DNMTs, Figure 2). The most frequently methylated nucleotides are cytosine residues located within CpG dinucleotides (Jin and Liu, 2018). They are usually found at the 5′ end of many gene regulatory regions but can extend into the exons (Deaton and Bird, 2011). CpG dinucleotides are not evenly distributed and can be categorized into dense CpG islands with CpG sequences or scattered out-of-island regions (Rodriguez-Rodero et al., 2017). In normal human somatic cells, 70%–90% of CpG dinucleotides are methylated (Ehrlich et al., 1982; Shaknovich et al., 2010). Cytosine methylation affects the expression of diabetes-related genes by changing the chromatin structure and altering the accessibility of transcription machinery. In addition to regulating the expression of various genes, methylation plays a vital role in cell differentiation and female X-chromosome inactivation (Shi et al., 2016). Repeating genomic sequences are extremely unstable, and their hypermethylation may prevent chromosomal instability, easy breakage, translocation, and gene disruption caused by the repeating DNA sequence, which can promote the expression of genes on that chromosome (Singh et al., 2011).

## DNA Methylation and Diabetic Cardiomyopathy

DNA methylation is an important epigenetic mechanism that controls cell differentiation and transcriptional potential in mammals. It regulates gene expression by inhibiting the binding of transcription factors to DNA. Many factors affect DNA methylation status, including the environment, diet, and aging. Recent studies have shown that abnormal DNA methylation is closely related to the occurrence and development of many cardiovascular diseases, such as coronary heart disease (Kim et al., 2010; Navas-Acien et al., 2021), atherosclerosis (AS) (Tao et al., 2021), hypertension (Dupriest et al., 2020; Amenyah et al., 2021), HF (Kao et al., 2010; Haas et al., 2013; Madsen et al., 2020), and DCM (Liu ZZ. et al., 2014; Guo et al., 2021).

Studies have shown that CpG islands are present in the promoter region of the sarcoplasmic reticulum Ca^2+^-ATPase (SERCA2a). In diabetic hyperglycemia, *in vivo* stimulation of cardiomyocytes with pro-inflammatory tumor necrosis factor-alpha (TNF-α: 50 ng/ml) increases methylation of the SERCA2a promoter region by increasing the level of DNA methyltransferase, which reduce the expression of SERCA2a (Kao et al., 2010). SERCA2a mediates the relaxation of the heart by transferring Ca^2+^ from the cells into the sarcoplasmic reticulum. Downregulation of SERCA2 expression can cause diastolic dysfunction and ultimately lead to the development of DCM. Changes in TNF-α expression in cardiomyocytes may underlie HF in diabetic patients since there is a positive correlation between HF severity and TNF-α (Asrih and Steffens, 2013). El-Osta *et al.* have shown that transient exposure of aortic endothelial cells to hyperglycemia induces sustained-onset epigenetic changes in the promoter of the NF-κB p65 subunit, which leads to increased expression of the p65 gene (El-Osta et al., 2008). Pirola *et al.* found that hyperglycemia can alter CpG methylation and, thus gene expression under diabetic conditions. A study reported that Keap1 protein expression was increased by demethylation of CpG islands in the promoter and reduced Nrf2 activity, thus inhibiting the transcription of various antioxidant genes and ultimately disrupting the redox balance in diabetes (Liu ZZ. et al., 2014). Activation of the renin-angiotensin-aldosterone system pathway plays a significant role in DCM. In DCM, genes in this pathway are typically upregulated, which eventually leads to myocardial hypertrophy. Bogdarina et al. showed that the proximal promoter of the angiotensin II type-1b (AT1b) gene is hypomethylated in the adrenal gland, and AT1b expression highly depends on promoter methylation *in vitro* (Bogdarina et al., 2007).

Hyperglycemia significantly affects human vascular chromatin and transcriptional upregulation of genes involved in metabolism and cardiovascular diseases (Pirola et al., 2011). The Epidemiology of Diabetes Interventions and Complications (EDIC) study examined DNA methylation profiles in whole blood isolated at baseline. The results indicate that changes in DNA methylation differences during diabetes persist at specific sites associated with blood glucose for several years. There was also evidence of persistent hypomethylation of the thioredoxin-interacting protein (TXNIP) gene associated with hyperglycemia and related complications (Chen et al., 2016). Recent studies identified that liver X receptor alpha (LXRα) is expressed in the myocardium of diabetic rats induced by streptozotocin (STZ). In addition, there are significant differences in the methylation status of the LXRα gene in the ventricles of control rats compared to the status in diabetic rats (Cheng et al., 2011).

A clinical study showed that age-related increases in methylation are negatively correlated with hepatic liver glucokinase (Gck) expression by studying the degree of Gck methylation and GcK expression in three age groups (Jiang et al., 2008). These results suggest that DNA methylation plays a significant role in increasing age-dependent insulin resistance and susceptibility to diabetes. Insulin resistance impairs heart contractility and increases oxidative stress, leading to cardiomyocyte apoptosis, myocardial fibrosis, remodeling, and cardiac hypertrophy, and thus resulting in DCM (Dobrin and Lebeche, 2010; Chemaly et al., 2011; Bobbert et al., 2012; Lebeche, 2015). Recent studies have shown that cardiac insulin resistance significantly contributes to the pathogenesis and progression of HF (Saotome et al., 2019).

JunD, a member of the activator protein 1 (AP-1) family transcription factors, stimulates or inhibits the expression of a variety of genes. JunD is under several layers of regulation, including transcriptional, post-transcriptional, protein post-translational modification, and protein-protein interactions. JunD is involved in the occurrence and development of DCM. Hussain et al. reported that the levels of JunD mRNA and protein are downregulated in the heart of patients with T2D and STZ-induced diabetic mice. JunD is epigenetically regulated by promoter hypermethylation, post-translational histone modifications, and miRNA-mediated translational repression by miR-673/menin axis. This indicates that multiple epigenetic mechanisms can synergize o alter gene expression rather than acting independently (Hussain et al., 2020). In addition, this cell-type-specific analysis revealed that gene programs associated with distinct biological processes are differentially regulated in diabetes.

Interestingly, despite these changes in gene expression, cell-type-specific DNA methylation signatures in genic and regulatory regions remain stable in diabetes. Analysis of heterocellular interactions in the diabetic heart suggests that the interplay between fibroblasts and monocytes is pivotal. A previous study showed that diabetes could change gene expression but not DNA methylation in cardiac cells (Lother et al., 2021).

Other regulatory factors are also involved in the occurrence and development of DCM. BRD4 is a member of the BET (bromodomain and extra-terminal domain) family of epigenetic regulators. High expression of BRD4 results in cardiac hypertrophy and plays an essential role in the pathogenesis of high glucose-induced cardiomyocyte hypertrophy through the AKT pathway in H9C2 cells and a diabetes rat model (Wang et al., 2019). In addition, JQ1 inhibits BRD4, improves mitochondrial function, and repairs cardiac structure and function by activating PINK1/Parkin-mediated mitophagy in high-fat diet-induced DCM (Mu et al., 2020).

In summary, these findings indicate that epigenetic marks such as DNA methylation play a significant role in DCM (Table 1).

**TABLE 1:** Summary of studies examining DNA methylation in DCM.

Species	Genes	Methylation status	Reference
DCM patients	RASSF1A	Hypermethylation	Tao et al. (2019)
STZ-induced diabetic rats	LXRα	Demethylation	Cheng et al. (2011)
Type 2 diabetes patients	Keap1	Demethylation	Li et al. (2015)
STZ-induced diabetic rats	AT1b	Undermethylated	Bogdarina et al. (2007)
Type 2 diabetes patients	HIF3A	DNA methylation	Guo et al. (2021)
db/db mice	Histone H3	H3K9 and H3K23 acetylation	Gaikwad et al. (2010)
		H3K4 dimethylation	
Diabetic rats	p21(Waf1/Cip1)	DNA methylation	Monkemann et al. (2002)
Type 2 diabetes patients and STZ-induced diabetic mice	JunD	Hypermethylation, Post-translational modification	Hussain et al. (2020)
STZ-induced diabetic mice	Sirt1 and DNMT3b	H3 acetylation and DNA Demethylation	Costantino et al. (2018)

STZ, streptozotocin.

## Histone Modification

Currently, there are 5 classes of histones identified in mammals: H1, H2A, H2B, H3, and H4. Histone modification mechanism. Histone modification is an epigenetic event that occurs via methylation, acetylation, phosphorylation, adenylation, ubiquitination, and ADP ribosylation (Bauer and Martin, 2017). These modifications affect the transcriptional activity of associated genes.

Covalent post-translational modification of histones can alter genome stability in response to changes in the environment, resulting in alterations to gene expression in pathological states such as metabolic stress. Along with CpG methylation, histone modifications control the accessibility of nucleosomes for transcription. Histone modifications also influence the binding capacity of other proteins to histones through changes in local hydrophobicity, RNA polymerase status, and binding affinity to other transcription coactivators. Various post-translational modifications can occur at the N-terminus of histones, including phosphorylation, acetylation, methylation, and ADP-ribosylation. It is challenging to decode specific post-translational modifications for individual histones or nucleosomes (e.g., location of nucleosomes regarding the gene transcriptional start site). However, histone modifications can communicate with each other. Factors including sites, types, and degrees of histone modifications contribute to the complexity of the histone code.

Histone acetylation and deacetylation, mediated through coactivator complexes containing histone acetyltransferases (HATs) and co-repressor complexes containing HDACs, respectively, represent the primary machineries controlling gene expression. HAT-mediated histone acetylation disengages intra- and inter-nucleosomal interactions to loosen the chromatin structure and turn on gene transcription. Histone changes through acetylation (attaching an acetyl group to lysine residues to neutralize its basic charge) or deacetylation using HDACs modulates the chromatin state (euchromatin is accessible whereas heterochromatin is inaccessible (Pasquier et al., 2015).

HDAC1 plays a significant role in the prevention and treatment of cardiac dysfunction. Resveratrol activates HDAC1 to prevent cardiomyocyte apoptosis and endoplasmic reticulum stress, reducing heart dysfunction in diabetic rats. SIRT1-dependent H3 deacetylation is also important to the response to myocardial injury and involves PERK/eIF2α, ATF6/CHOP, and IRE1α/JNK (Guo et al., 2015). The downregulation of Sirt1 and DNMT3b induced by diabetes promotes H3 acetylation and DNA demethylation of the p66Shc promoter, leading to DCM (Costantino et al., 2018). In addition, a recent study on obese mice reported that the lack of cardiac mitochondrial acetaldehyde dehydrogenase 2 affected the epigenetic SUV39H-SIRT1 loop, resulting in changes in transcription, autophagy, and myocardial metabolism (Wang et al., 2018).

We often refer to this relationship between glycemic control and the development of organ dysfunction as “blood sugar memory.” Unstable blood glucose can lead to DCM (Pepin and Wende, 2019) due to a combination of the release of ROS from chronic exposure to hyperglycemia (Lebeche, 2015), histone H3K4 methylation, and epigenetic activation of NF-κB-p65 in aortic vascular endothelial cells (El-Osta et al., 2008), and eventually lead to DCM. El-Osta et al. further studied the expression of the p65 gene and found that in transient hyperglycemic conditions, glucose inhibited the H3K9me2 and H3K9me3 marks on the p65 promoter and promoted the H3K4me1 mark (Brasacchio et al., 2009). Furthermore, histone lysine methyltransferase, SET7/9 (a novel coactivator of NF-κB), can target histone H3K4, enhance its methylation, and increase in NF-κB expression through histone methylation. Studies have shown that NF-κB-p65 is involved in the pathogenesis of pathological cardiac hypertrophy (Xu et al., 2015). Moreover, telmisartan and esculetin attenuate increases in histone modifications, such as H3K9me2, H3K9Ac, H2AK119Ub, and H2BK120Ub in the heart of T2D rats and ameliorate type 2 DCM by reversing H3, H2A, and H2B histone modifications (Kadakol et al., 2017). This suggests that histone modifications induced by exposure to high blood glucose concentrations play an essential role in DCM.

Histone dysregulation caused by environmental factors can synergize with hyperglycemia to induce diabetic complications. Gaikwad *et al.* reported a metabolic abnormality associated with renal failure in diabetic nephropathy. Renal metabolic disorders alter histone H3 acetylation in diabetic mice, further damaging myocardial cells. Examining the cross-sections of the hearts of the diabetic mice demonstrated that renal failure increases myocardial disease-related gene expression and cardiomyocyte hypertrophy (one characteristic of DCM) rather than cardiomyocyte proliferation (Gaikwad et al., 2010). Cardiac histone H3 modification caused by diabetic nephropathy thus plays a significant role in driving DCM.

The role of HDACs in cardiac hypertrophy and failure is complex, with some displaying antihypertrophic properties, whereas others exhibit pro-hypertrophic features. Recent studies have shown that HDACs can improve myocardial function and inhibit cardiac remodeling in DCM, as HDACs can improve cardiac function and inhibit myocardial remodeling in diabetic hearts (Chen et al., 2015). Kronlage et al. showed that O-GlcNAcylation of HDAC4 alleviates HF in diabetes and found that O-GlcNAcylation of HDAC4 at serine (Ser)-642 is cardioprotective in DCM and inhibits Ca^2+^/calmodulin-dependent protein kinase II signaling (Kronlage et al., 2019).

Taken together, these studies suggest that histone acetylation plays a significant role in regulating gene expression associated with diabetic complications, including DCM. In diabetes, endothelial cells are exposed to hyperglycemia, and histone acetylation is increased in the promoters of crucial genes in the extracellular matrix, leading to increased human p300 (Mathiyalagan et al., 2010), vascular diabetic complications and cardiomyocyte hypertrophy.

In addition, histone modification affects blood glucose memory in cardiomyocytes, mainly through changes in the expression of important genes.

## Non-Coding RNAs and Diabetic Cardiomyopathy

Accumulating evidence suggests that pathological hypertrophy and cardiac remodeling may contribute to DCM. Non-coding RNAs, including long non-coding RNAs (lncRNAs) and small non-coding RNAs (e.g., miRNAs, short-interfering RNA, and piwi-interacting RNAs), play important roles in DCM. MiRNAs are ubiquitous in eukaryotic cells and are about 21–23 nucleotides in length. As endogenous post-translational inhibitors, miRNAs bind to the 3’end of complementary mRNA to degrade the mRNA or inhibit translation, thereby reducing the expression of the target gene. lncRNA usually regulates protein through processes such as competitive inhibition and recruitment. They also participate in the entire transcription process and play important roles as scaffolds that provide platforms for the interaction between chromatin modification complexes and transcription complexes.

In recent years, small RNAs, especially miRNAs, have gained increasing attention in cardiovascular disease studies. Exploring the regulatory importance of small RNAs in DCM will facilitate the development of new DCM therapeutic.

## Small RNAs in the Pathological Development of Diabetic Cardiomyopathy

Small RNAs negatively modulate gene expression, primarily through binding to the target mRNA and subsequently inducing their degradation or suppressing translation. In short, small RNAs can regulate DCM pathogenesis through the aggravation of myocardial fibrosis, oxidative stress, apoptosis, cardiac electrical remodeling, or epigenetic modification (Table 2). Recent studies have identified that miRNAs play a vital role in the etiology of DCM (Guo and Nair, 2017; Xia and Song, 2020). By upregulating or down-regulating different target genes, the same miRNA can play many roles in cardiomyocyte or myocardial fiber pathology (Xia and Song, 2020). Feng et al. reported that miR-133a was downregulated in hypertrophic cardiac tissue under high glucose conditions, and miR-133a overexpression prevented hypertrophic changes in cardiomyocytes (Feng et al., 2010). Different miRNAs can also synergistically regulate DCM by upregulating or down-regulating target genes (Xia and Song, 2020). According to previous reports, multiple miRNAs, including miR-1 (Ikeda et al., 2009), miR-133a (Zhu Y.-F. et al., 2021), miR-351 (Zhu Y.-F. et al., 2021), miR-199a (Li et al.,2017), and miR-451 (Kuwabara et al., 2015), regulate cardiac hypertrophy in DCM by upregulating or down-regulating target genes. In addition, some miRNAs only alter the levels of one target gene under high-glucose conditions. They achieve DCM pathological changes by singly up- or down-regulating target genes under high glucose conditions. Liang et al. revealed that knockdown of miR-451 attenuated cardiac fibrosis and improved cardiac function by suppressing the endothelial-to-mesenchymal transition (EndMT) in diabetic mouse hearts (Liang et al., 2019).

**TABLE 2:** Summary of miRNAs involved in the pathogenesis of DCM.

Mechanism	miRNAs	Regulated genes	Species	Reference
Hypertrophy	↓miR-1	MEF2a/Gata4	HG-treated NRCMs and diabetic rats	Ikeda et al. (2009)
	↓miRNA-146a	DLST	HG-treated NRCMs	Heggermont et al. (2017)
	↓miR-133a	SCK1/IGF1R	STZ-induced diabetic mice	Feng et al. (2010)
	↓miR-150	p300	Diabetic rats	Duan et al. (2013)
	↑miR-200c	DUSP-1	HG and STZ-induced diabetic rats	Singh et al. (2017)
Fibrosis	↓miR-152-3p	Wnt1/β-catenin	HG-treated NRCMs	Xu et al. (2021)
	↑miR-26a/b-5p	GAS5	STZ-induced diabetes model	Zhu et al. (2021a)
	↓miR-29b-3p	circHIPK3	STZ-induced diabetes model	Wang et al. (2021)
	↓miR-223	NLRP3	HG-induced cardiomyocyte injury mode	Xu et al. (2020)
	↓miR-155	TGF-β1	STZ-induced diabetes model	Zhang et al. (2016)
	↓miR-21	DUSP8	HG-induced diabetes model	Liu et al. (2014a)
Apoptosis	↑miR-133a	caspase-3, caspase-8	Type 2 diabetic rats	Habibi et al. (2020)
	↑miR-181a-5p	JAK2/STAT3	Human cardiomyocytes	Tan et al. (2021)
	↑miR-146a	MAPK	H9C2 cells	Chu et al. (2021)
	↑miR-140-5p	HDAC4/Neat1	STZ-induced diabetic mice	Zou et al. (2019)
	↓miR-1	IGF1	Diabetic rats	Delfan et al. (2020)
	↓miR-675	VDAC1	STZ-induced diabetic rats	Li et al. (2016b)
Autophagy	↑miR-221-3p	GAS5	STZ-induced diabetic rats and HG treated H9C2	Chen and Zhang, (2021)
	↑miR-551b-5p	DCRF	STZ-induced diabetic rats	Feng et al. (2019)
	↑miR-34a	Bcl2 and Sirt1	HG treated H9C2	Zhu et al. (2019)
		-	STZ-induced diabetic mice and HG- induced cardiomyocytes	Ni et al. (2020)
Oxidative stress	↓miR-128	PIK3R1/Akt/mTOR	C57 BL/6 mice	Zhan et al. (2021)
	↓MiR-22	Sirt1	HFD and STZ-induced diabetic mice	Tang et al. (2018)
	↓miR-1 and ↓miR-499	RyR2	Diabetic rats	Yildirim et al. (2013)
	↓miR-150	P300	Diabetic rats	Duan et al. (2013)
Inflammation	↓miR-130	PPAR-γ	H9C2 cells	Chu et al. (2018)
	↑miR-150-5p	Smad7	HG- induced diabetes model	Che et al. (2020)
	↓miR146a	IL6, TNFα, IL-1β, MCP-1	Human cardiac microvascular endothelial cells and STZ-induced diabetes	Feng et al. (2017)
	↓miR-214-3p	KCNQ1	STZ-induced diabetes model and cardiomyocytes treated with HG	Yang et al. (2018)
	↓miR-675	VDAC1	STZ induced diabetic rats	Li et al. (2016b)

NRCMs, neonatal rat cardiomyocytes; HFD, high fat diet; HG, high glucose ; STZ, streptozotocin.

## Clinical Application of Small RNAs in Diabetic Cardiomyopathy

The above findings reveal a novel mechanism of DCM pathogenesis involving small RNAs, provide new strategies for the clinical diagnosis, treatment, or prevention of DCM, and highlight potential strategies for the development of small RNA-based therapies to treat diabetes-related cardiovascular complications. Li et al. demonstrated that inhibiting miR-320 could rescue DCM in diabetic mice (Li et al., 2019). This suggests that targeting miR-320 may represent a potential therapeutic strategy to treat diabetes-induced cardiac dysfunction. A recent study has identified miRNA-497 as a potential therapeutic agent for diabetic wound healing, owing to its repressive effect on pro-inflammatory cytokines (Ban et al., 2020). Furthermore, some studies indicate that as circulating miRNAs can be altered depending on the phase of the disease, they could also be used as potential biomarkers for assessing the development and progression of DCM. This also means that early intervention can prevent severe complications in DCM (Guo and Nair, 2017). In addition, other small RNAs, such as piwi-interacting RNAs, could provide therapeutic targets for the treatment of pathological hypertrophy and maladaptive cardiac remodeling (Gao et al., 2020).

## Conclusion

In summary, epigenetics is an exciting and promising emerging research field in cardiovascular research and therapy. Achieving a better understanding of the roles played by epigenetics in DCM is an important strategy to improve early diagnosis and treatment of DCM, as well as the establishment of new therapeutics. In addition, drugs such as methylation and acetylation inhibitors represent promising candidates for targeted DCM therapies, and tissue-specific epigenetic modifier drugs may offer a fresh therapeutic perspective for DCM patients.

## Abbreviations

AP-1, activator protein 1; AT1b, angiotensin II type 1b; ATF6: activating transcription factor 6; AKT/PKB, protein kinase B; BET, bromodomain and extra-terminal domain; BRD4, bromodomain-containing protein 4; CHOP, transcription factor C/EBP homologous protein; DCM, diabetic cardiomyopathy; DLST, dihydrolipoamide S-succinyltransferase; DNMTs, DNA methyltransferases; DNMT3b, DNA methyltransferase 3b; DUSP-1: dual specificity phosphatase 1; DUSP-8, dual specificity phosphatase 8; eIF2α, eukaryotic initiation factor 2 alpha; EndMT, endothelial-to-mesenchymal transition; GAS5, growth arrest-specific transcript 5; Gck, glucokinase; HAT, histone acetyltransferase; HDAC, histone deacetylase; HF, heart failure; HIF3A, hypoxia-inducible factor 3alpha; IRE1α, inositol-requiring protein 1alpha; IGF1R, insulin-like growth factor 1 receptor; JAK2, janus kinase 2; Keap1, Kelch-like ECH-associated protein 1; LXRα, liver X receptor α; MAPK, mitogen-activated protein kinases; Mef2a, myocyte enhancer factor 2a; NF-κB, nuclear factor kappa-light-chain-enhancer of activated B cells; NLRP3: nod-like receptor protein 3; Nrf2, nuclear factor erythroid 2-related factor 2; PERK, Protein kinase RNA-like endoplasmic reticulum kinase; PPAR-γ, peroxisome proliferative activated receptor, gamma; RASSF1A, ras association domain family protein1 isoform A; RyR2, ryanodine receptor 2; SERCA2a, sarcoplasmic reticulum Ca2+-ATPase 2a; Sirt1, sirtuin 1; Smad7, SMAD family member 7; STAT3, signal transducer and activator of transcription 3; SUV39H1, suppresssor of variegation 3-9 homolog; T2D, type 2 diabetes; TNFα, tumor necrosis factor-alpha; TXNIP, thioredoxin-interacting protein; VDAC 1, voltage-dependent anion channel 1.
